# SOX14 hypermethylation as a tumour biomarker in cervical cancer

**DOI:** 10.1186/s12885-021-08406-2

**Published:** 2021-06-07

**Authors:** Jing Zhao, Huiling Cao, Wenfan Zhang, Yongjuan Fan, Shujuan Shi, Rong Wang

**Affiliations:** 1 Department of Gynecology and Obstetrics, Tianjin Medical University General Hospital, Tianjin, China; 2 Department of Laboratory Medicine, Tianjin Medical University, Tianjin, China; 3 Department of Gynecology and Obstetrics, Tianjin First Central Hospital, Tianjin, China; 4 Department of Human Anatomy and Histology, Tianjin Medical University, Tianjin, China

**Keywords:** Cervical cancer, SOX14, DNA methylation, Methylation-specific PCR (MSP), Quantitative methylation-specific PCR (QMSP)

## Abstract

**Background:**

The association between SOX14 and cancer has been reported. The aim of this study was to identify and validate the potential value of SOX14 methylation in the early detection of cervical cancer.

**Methods:**

First, we extracted the data for SOX14 methylation and expression within cervical cancer from The Cancer Genome Atlas (TCGA) database and analysed them via UALCAN, Wanderer, MEXPRESS and LinkedOmics. Subsequently, according to the bioinformatics findings, primers and probes were designed for the most significantly differentiated methylation CpG site and synthesized for methylation-specific PCR (MSP) and quantitative methylation-specific PCR (QMSP) to verify SOX14 methylation in both cervical tissuses and liquid-based cell samples. Eventually, the clinical diagnostic efficacy of SOX14 methylation in the normal, cervical intraepithelial neoplasia, and cancer groups was analysed by ROC^AUC^.

**Results:**

Pooled analysis demonstrated that SOX14 methylation levels were significantly increased in cervical squamous cell carcinoma and endocervical adenocarcinoma (CESC) compared to normal tissues (*P* < 0.001). Both the verification and validation cohorts indicated that the methylation level and the positive rate of SOX14 gradually increased with increasing severity from normal to cancer samples (*P* < 0.01). When the cut-off value was set as 128.45, the sensitivity and specificity of SOX14 hypermethylation in the diagnosis of cervical cancer were 94.12 and 86.46%, respectively. When taken as a screening biomarker (>CINII), the sensitivity was 74.42% and the specificity was 81.48%, with a cut-off value of 10.37.

**Conclusion:**

SOX14 hypermethylation is associated with cervical cancer and has the potential to be a molecular biomarker for the screening and early diagnosis of cervical cancer.

**Supplementary Information:**

The online version contains supplementary material available at 10.1186/s12885-021-08406-2.

## Background

Cervical cancer ranks as the fourth most frequently diagnosed cancer and the fourth leading cause of cancer death among women, with an estimated 604,000 cases and 342,000 deaths worldwide in 2020 [1]. It is the second most common type of cancer in women in the Southeast Asia region and a major cause of cancer death among women in low- and middle-income countries (LMICs) [2]. Nevertheless, in some of the wealthier countries of Central and Eastern Europe, screening has effectively reduced the incidence and mortality of cervical cancer among women < 50 years old [3].

Currently, the Pap smear, the ThinPrep cytologic test (TCT), and human papillomavirus (HPV) testing are the most commonly used methods for cervical cancer screening [4]. However, in addition to the lower sensitivity of cytology methods (Pap smear and TCT), the results of the different screening infrastructures vary widely as does the labour intensiveness of these methods [5, 6]. Regarding HPV tests, despite the superior sensitivity, the lower specificity is an inevitable problem leading to a need to identify innovative biomarkers for cervical cancer screening [7]. Studies have revealed that DNA hypermethylation in the tumour suppressor gene promoter region is an early event in human cancers and is negatively correlated with gene expression [8]. Additionally, most of the techniques on DNA methylation detection are generally easy to perform, less labour intensive and repeatable. For those reasons, the identification and performance of cancer-related DNA hypermethylation biomarkers in clinical settings have attracted the attention of clinical practitioners and researchers.

SOX14 is a member of the SOX gene family, which mediates the binding of high-mobility group (HMG) domains to DNA and has regulatory functions in development, the cell cycle and differentiation [9]. Previous studies have discovered that many genes in the SOX family participate in carcinogenesis, such as SOX1, which can affect the growth and invasion of cancer cells in cervical cancer [10], breast cancer [11], lung cancer [12], glioblastoma [13] and nasopharyngeal cancer [14]. SOX2, SOX6 and SOX17 are associated with the occurrence of sarcomas [15]. SOX10 has been linked to melanoma metastasis [16], and SOX7 to acute myeloid leukaemia [17]. Recently, Jiali Hu et al. emphasized the critical roles of the SOX gene family as regulators in the progression of gynaecological cancers [18]. There is potential for gynaecologists to use SOX genes to make precise clinical decisions [18]. Furthermore, aberrant methylation of SOX genes in cancer has been frequently reported; for instance, SOX1 and SOX11 present hypermethylation in cervical cancer and endometrial cancer, respectively. However, hypomethylation in the SOX9 promoter, which increases SOX9 expression in prostate cancer, has also been reported [19].

SOX14 is also involved in the development of tumours. Deb S et al. found that SOX14 can induce apoptosis of cervical cancer cell lines by activating the p53 pathway [20]. Li F et al. showed that SOX14 promotes the proliferation and invasion of cervical cancer cells through the Wnt/β-catenin pathway [21]. In addition, analysis of the genome-wide DNA methylation map of chronic lymphocytic leukaemia showed that SOX14 was one of the methylated genes in patients with chronic lymphocytic leukaemia [22].

Therefore, the aims of this study were to 1) further explore the relationship of SOX14 methylation in cervical cancer using integrated datasets and web tools and 2) validate the potential value of SOX14 methylation in the screening and early diagnosis of cervical cancer.

## Methods

### Pooled analysis

Datasets of DNA methylation was extracted from the TCGA database and included clinical information from cervical tissues (Table S1). The methylation level of SOX14 in normal and primary tumour patients with cervical squamous cell carcinoma and endocervical adenocarcinoma (CESC) was comparatively analysed using the UALCAN web tool (http://ualcan.path.uab.edu/index.html) [23]. The mean SOX14 methylation level in each CpG site in CESC was generated with Wanderer (http://maplab.imppc.org/wanderer/) [24]. The raw expression data were collected from TCGA& GTEx and analysed by GEPIA(http://gepia.cancer-pku.cn/) [25]. The association of SOX14 mean methylation and expression in CESC was compared via MEXPRESS (https://mexpress.be/) [26]. The association of SOX14 methylation and CESC clinical information was analysed using the LinkedOmics tool (http://www.linkedomics.org/login.php) [27].

### Sample collection

Cervical frozen tissues and liquid-based cell specimens were available from the Department of Gynecology and Obstetrics, Tianjin Medical University General Hospital, and Tianjin First Central Hospital from January 2016 to June 2017. No patients included had a history of hysterectomy, radiotherapy or chemotherapy and a history of taking immunosuppressive agents or other tumours. Moreover, none of the patients involved were pregnant. Normal tissue samples and normal TCT samples from benign disease patients with fibroids, uterine prolapse, hypermenorrhoea, etc.

All the samples were collected before treatment and a diagnosis was made by experienced gynaecological pathologists, with histological classification as the reference; the samples were classified as normal, cervical intraepithelial neoplasia II (CINII), CINIII or cancer. This study was approved by the Medical Ethics Committee of Tianjin Medical University, and all the patients gave informed consent.

A total of 36 tissue specimens and 113 TCT specimens were collected for the tests. The frozen tissue samples consisted of 19 normal samples, and 17 cancer samples stored at − 80 °C. The TCT specimens included 27 normal cervical samples (median age: 43 years), 36 CINII stage specimens (median patient age: 42 years), 33 CINIII stage specimens (median patient age: 40 years), and 17 cervical cancer specimens (median patient age: 54 years). The FIGO staging of cervical cancer results were as follows: 6 cases of IA1, 3 cases of IB1, 1 case of IB2, 4 cases of IIA, 2 cases of IIB, and 1 case of IIIB. The specimens were obtained by clinicians using a disposable cervical specimen collection brush, stored in TCT preservation solution (SumDod, Guangzhou, China), and stored at 4 °C.

### DNA extraction and quality testing

Genomic DNA from frozen tissues was extracted using a TIANamp Genomic DNA Kit (Tiangen Biotech, Beijing, China) according to the manufacturer’s instructions. DNA from cervical liquid-based cell specimens was isolated by phenol/chloroform extraction [28]. DNA concentration and absorbance (A260/280) were measured using a Nanodrop 2000c spectrophotometer (Thermo Fisher Scientific, Waltham, MA, USA). DNA samples with concentrations ≥100 ng/μl and A260/280 ratios of approximately 1.8 were analysed. To ensure that the size of the obtained DNA fragment was intact for the subsequent Methylation-specific PCR (MSP), ladder PCR was performed as previously described [29]. A full-length gel picture was presented in Figure S1.

### Bisulfite treatment

One microgram of genomic DNA per sample was modified using the EZ DNA methylation kit (Zymo Research Corp, Irvine, US) according to the manufacturer’s instructions. Leukocyte DNA from healthy women was used as a negative control for methylation, while in vitro methylated leukocyte DNA produced using M. SssI methyltransferase (New England Biolabs, Ipswitch, USA) was used as a positive control.

### Methylation-specific PCR (MSP)

Methylated primers were designed using Methyl Primer Express v1.0 and synthesized by Sangon Biotech (Table 1). Each reaction was performed in a total reaction volume of 30 μl, containing 1.8 μl MSP primer mix (10 μM), 1.5 μl bisulfite-treated DNA, 0.6 μl dNTPs (10 mM), and 0.5 U AmpliTaq Gold DNA polymerase. The MSP thermal cycling program was as follows: 10 min at 95 °C; 95 °C for 60 s, 55 °C for 60 s, and 72 °C for 60 s, for a total of 40 cycles; and a final elongation step of 7 min at 72 °C. Leukocyte (leu) DNA from healthy women was used as a negative control, and in vitro methylated (iv) leukocyte DNA was used as a positive control for each MSP.

**Table 1:** Primers and probes used in MSP and QMSP

	Primers or Probes	Sequence(5′ to 3′)	Size (bp)
**MSP**	SOX14-methylation primer	F:GTTCGTGGGGGTTTTCGAC	85
		R:CAAAAAATAAAACGCCGAAACCG	
	SOX14-unmethylation Primer	F:GTTTGTTTGTGGGGGTTTTTGATG	94
		R:TCCAACAAAAAATAAAACACCAAAACCA	
**QMSP**	SOX14 primer	F: GTTCGTGGGGGTTTTCGAC	85
		R: CAAAAAATAAAACGCCGAAACCG	
	SOX14 probe	6-FAM-TGAGCGCGTTCGAGAAAGTTCGGG-BHQ1	
	ACTB primer	F: TGGTGATGGAGGAGGTTTAGTAAGT	100
		R: AACCAATAAAACCTACTCCTCCCTTAA	
	ACTB probe	6-FAM-ACCACCACCCAACACACAATAACAAACACA-BHQ1	

### Quantitative methylation-specific PCR (QMSP)

The methylated primers for QMSP were the same as those used for MSP. The probes were designed using Cone Manager 9.0 software and synthesized by Sangon Biotech. The ACTB gene was used as a methylation reference gene. Each reaction was performed in a total 10 μl reaction volume containing 5 μl 2 × Master Mix, 2.5 μl BS-DNA (10 ng/μl), forward and reverse primer (10 μmol/L) at 0.3 μl each, probe (5 μmol/L) at 0.4 μl, and ddH_2_O at 1.5 μl. Three wells were set in each DNA sample. PCR was performed on an ABI PRISM® 7900HT Sequence Detection System. The QMSP thermal cycling program was as follows: 95 °C for 10 min followed by 50 cycles of 95 °C for 15 s and 60 °C for 1 min. The criteria for the interpretation of positive methylation results were as follows: Ct value < 50 (at least 2 of 3 multiple wells) with sufficient methylated DNA (200 pg DNA). The relative level of SOX14 methylation was expressed as (the average quantity of methylated SOX14 /the average quantity of ACTB) × 10,000 [30].

### Statistical analysis

Statistical analysis were performed using IBM SPSS 25.0 (IBM Corporation, New York, USA) and GraphPad Prism 8 (GraphPad Software, USA). The association between methylation in cg4945331 and expression from TCGA was analysis by Spearman. Kruskal-Wallis test was used to compare methylation levels and clinical stages. The threshold of each two groups was defined with the maximum value of the Youden index (YI), and the sensitivity, specificity were calculated accordingly. GraphPad Prism version 8.0 was used for statistically significant differences between two stages by the Mann–Whitney test. A *P*-value of less than 0.05 was considered statistically significant.

## Results

### Pooled dataset analysis

**Fig. 1:**
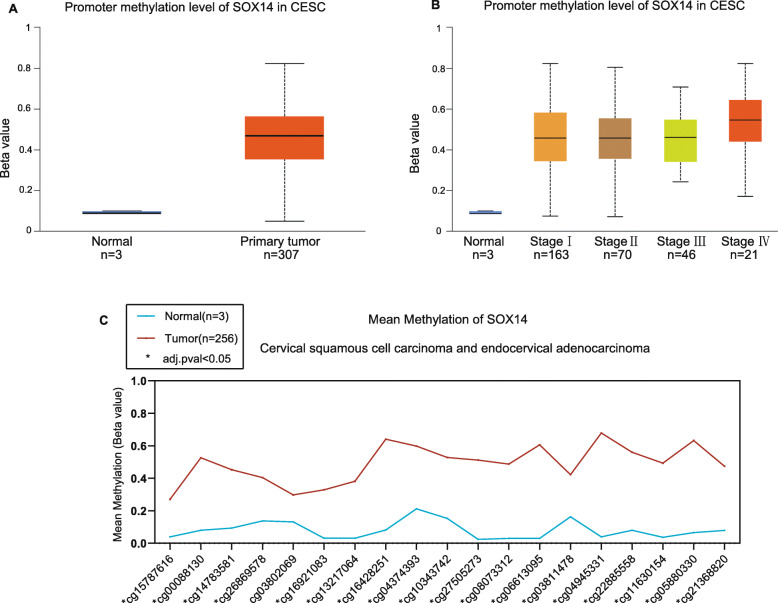
SOX14 promoter methylation level in CSEC. **(a)** SOX14 promoter methylation level in normal and cancer tissue. **(b)** SOX14 promoter methylation level increased as the FIGO stage rising from stage I to IV. The beta value indicates the level of DNA methylation ranging from 0 (unmethylated) to 1 (fully methylated). Box plot and *P* value of **A** and **B** were generated from UALCAN. **(c)** The levels of mean SOX14 DNA methylation in every CpG sites. Green font represents CpG islands,blue and red represent normal and tumor, respcetively; * represents adjusted *P*-value< 0.05. Plot and *P*-values were produced via Wanderer

First, the methylation level of SOX14 was significantly higher than that in normal tissues from UALCAN (*P* < 0.001). Furthermore, the methylation level was increased with tumour stage, and that of stage IV was the highest (*P* < 0.001) (Fig. 1a.b). The dataset from Wanderer demonstrated that the mean methylation level of each CpG site in the whole SOX14 gene (total of 19 CpG sites) was significantly increased in CESC compared to normal tissues (Fig. 1c) (*P* < 0.05). The most differential methylated CpG site was cg4945331 (Table S2).

**Fig. 2:**
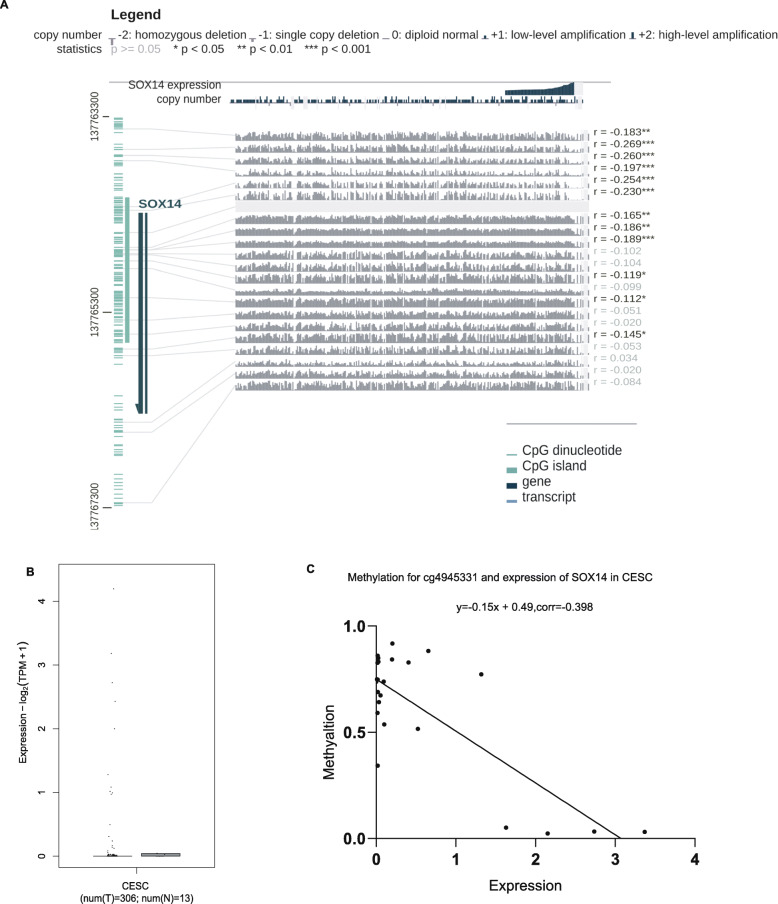
Association of SOX14 methylation and expression in CESC based on TCGA database. **(a)** The relationship between SOX14 expression and its methylation in 317 patients using MEXPRESS in different CpG site. **(b)** The expression of SOX14 in cevical tissues via GEPIA based on TCGA and GTEx. **(c)** Visualization the relationship of SOX14 expression and its methylation levels in cg4945331 was analysed based on TCGA database

From MEXPRESS, a total of 12 CpG sites, including cg4945331, were found to be significantly correlated with expression (Pearson correlation coefficients from − 0.112 to − 0.269) (Fig. 2a). However, SOX14 did not show any expression in normal cervix uterine from the GTEx (Fig. S2). Fig. 2b from GEPIA illustrated that only a few of cervical cancer samples with the expression. Hence, we analysed the raw expression data from TCGA, there were total of 25 cases with both methylation and expression levels, and further calculated the association between them. It was showed that the level of SOX14 methylation in cg4945331 was inversely correlated with SOX14 mRNA expression (corr = − 0.398 *P* < 0.05) (Fig. 2c), which was consistent with MEXPRE.

**Fig. 3:**
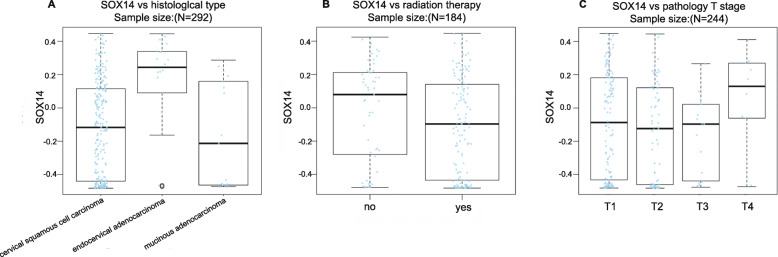
Association of SOX14 methylation and pathological features in CESC. **(a)** SOX14 methylation level is associated with histological type (*P* < 0.01), which higher in endocervical type of adenocarcinoma by Kruskal-Wallis Test. **(b)** SOX14 methylation level shows no difference with radiation therapy (*P* = 0.084, Wilcox Test), and **(c)** T stage (*P* = 0.185,Kruskal-Wallis Test)

Additionally, LinkedOmics found that the SOX14 methylation level was linked with the histological type (*P* < 0.01, *n* = 292). (Fig. 3a) It was higher in endocervical adenocarcinoma than in cervical squamous cell carcinoma and mucinous adenocarcinoma. However, SOX14 methylation had no significant relationship with radiation therapy (*P* = 0.084, *n* = 184) or T stage (*P* = 0.185, *n* = 244) (Fig. 3b,c).

### Verification of SOX14 methylation in cervical tissues using MSP

**Fig. 4:**
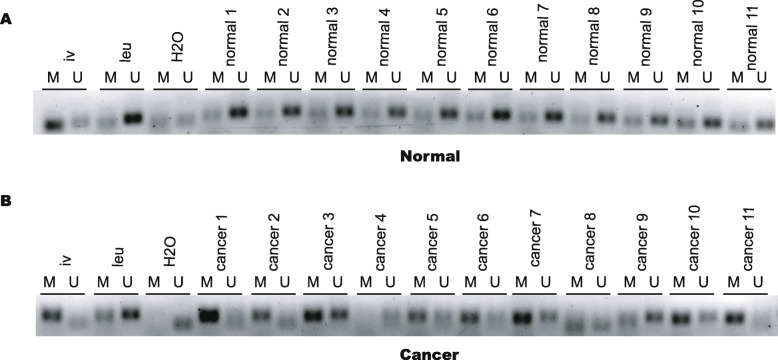
Verification of SOX14 methylation in cervical tissues using MSP. **(a)** The methylation positive rate in cancer tissues was 70.59% (12/17). **(b)** The methylation positive rate of SOX14 in the normal tissues was 5.26%(1/19). M:methylation, U: unmethylation; leu: Leukocyte DNA from healthy women was used as a negative control of methylation. iv: In vitro methylated leukocyte DNA was used as a positive control. Methylation positive results:the methylated belts were more heavy than the unmethylated belts. Methylation negative results:the methylated bands were lighter than the unmethylated bands. Full-length gels were presented in Supplementary Fig. S3

We performed MSP with primer sets targeting the sequence including the cg4945331 site with the top differential value (Table S2) to detect SOX14 hypermethylation in cervical tissues. Consistent with the bioinformatics results, the methylation positive rate in cancer tissues was 70.59% (12/17), which was higher than that in normal tissues (5.26%, 1/19) (Fig. 4). Full-length gel pictures were presented in Supplementary Fig S3 and Fig S4.

### Validation of SOX14 methylation in cervical liquid-based cells using QMSP

**Fig. 5:**
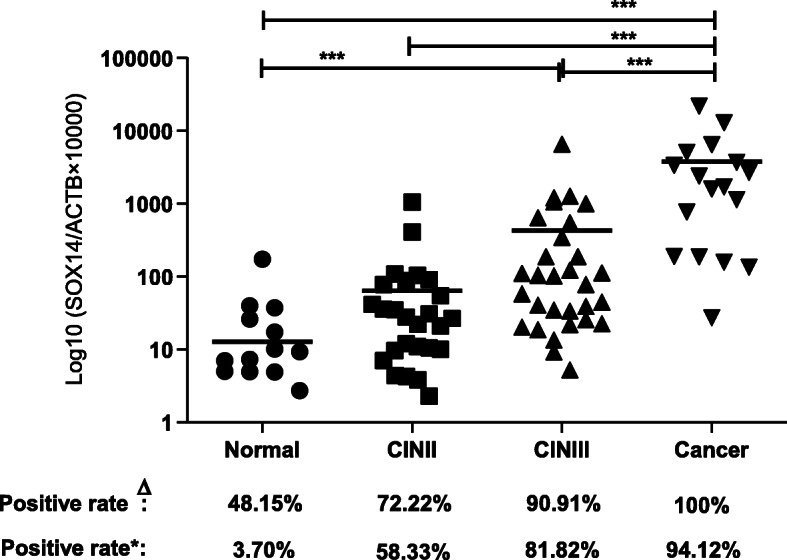
Validation SOX14 methylation in cervical liquid-based cells using QMSP. **(a)** Both the Positive rate and the methylation level of SOX14 increased with the severity of cervical lesions (* * * *P* < 0.0001, * *P* < 0.05). ^Δ^:represent the positive rate calculated directly from the value of QMSP.*: was to recalculate the positive rate according to the cut-off value (87.21)

Figure 5 illustrates that the methylation level of SOX14 increased with the severity of cervical lesions (H = 52.55, *P* < 0.01) (Table 2). Paired comparisons between groups showed a significant difference between each pair of groups (*P* < 0.05) (Table 2). Similarly, the SOX14 methylation positive rate increased from the normal to cancer samples and were 48.15% (13/27) in the normal samples, 72.22% (26/36) in CINII, 90.91% (30/33) in CINIII, and 100% (17/17) in cancer, respectively (Fig. 5). Despite a slightly higher methylation positive rate in normal cells, it was easy to determine an optimal threshold to discriminate each group since the quartile QMSP value was significantly different. As Table 3 and figure showed, with the optimal cut-off value of 87.21, the SOX14 methylation positive rate was 3.70% (1/27) in the normal group, 58.33% (21/36) in the CINII group, 81.81% (27/33) in the CINIII group, and 94.12% (16/17) in the cancer group.

**Table 2:** The comparison of QMSP value in each stage

Group	N	QMSP value [M (P25, P75)]
Normal	27	0.00 (0.00–9.38)
CIN II	36	10.71 (0.00–40.27)
CIN III	33	58.08 (21.08–267.19) ^*^
Cervical Cancer	17	1707.93 (187.79–4394.02) ^# ψ Δ^

^*^CIN III was compared with normal group (*P* < 0.001). Cancer was compared with the normal group^#^ (*P* < 0.001), CIN II ^ψ^ (*P* < 0.001), and CIN III^Δ^ (*P* < 0.001)

**Table 3:** Clinical evaluation of SOX14 methylation in each of the differentiated groups

Groups	AUC	Cut off	Sen (%)	Spe (%)	YI
Normal vs. Cancer	0.99	87.21	94.12	96.30	0.90
Precancer (Normal+CINII+CINIII) vs. Cancer	0.94	128.45	94.12	86.46	0.81
Normal vs. ≥ CINII	0.81	10.37	74.42	81.48	0.56

*AUC* Area Under Curve;Sen:sensitivity;Spe:specificity; *YI *Youden index

### Clinical evaluation of SOX14 hypermethylation in cervical cancer

The sensitivity and specificity between the groups were analysed according to ROC curves (Fig. S5). The highest ROC^AUC^ was 0.99 in the normal and cancer tissues, and the sensitivity and specificity in the diagnosis of cancer were 94.12 and 96.3%, respectively, with the optimal cut-off value (87.21) (Table 3). Since DNA methylation biomarkers were reported in a stage-specific manner, we further combined the precancer stage (normal & CINII & CINIII) and compared that combination with the cancer stage. Table 3 showed that the ROC^AUC^ had a sensitivity of 94.12% and specificity of 86.46%. The YI was 0.81, which indicated that SOX14 was a cancer-related biomarker. In addition, CINII is the split stage in the clinical screening programs, and we combined the CINII and worse stages (≥CINII) into a disease group. Table 3 showed that the ROC^AUC^ was 0.81, the sensitivity (74.42%) was decreased, but the specificity (81.48%) were comparable.

## Discussion

As a common malignancy of the female reproductive system, cervical carcinogenesis is a complicated process with multiple stages and steps. There is a gradual development process from precancerous lesions to cervical cancer, and early detection of cervical lesions is particularly important in clinical practice. Aberrant DNA methylation is an epigenetic hallmark of cancer and is known to play an important role in tumorigenesis and progression. Meanwhile, with the advancement of bioinformatics technology, it is feasible to identify novel methylation biomarkers via integrative multi-web tools [31–33]. Definitely, before these markers can be used clinically to benefit patients, a series of worldwide clinical evaluation studies are needed.

Hence, to further reveal the role of SOX14 methylation in cervical cancer and validate the potential clinical value of SOX14 methylation in the diagnosis of cervical cancer, we combined information from databases and clinical samples. Based on an integrative multi-web tool approach, we found that SOX14 was hypermethylated in CESC and correlated with its expression. Clearly, these findings indicate that SOX14 methylation participates in carcinogenesis by perturbing SOX14 gene regulation. More exciting, it was feasible for us to select a CpG site with the most differentiated methylation level between normal and cancer samples to design primers and probes for subsequent verification and validation via the Wander web tool. As expected, the MSP results demonstrated that the methylation positive rate of SOX14 in cancer tissues (70.59%) was obviously higher than that in normal tissues (5.26%). Thus, the biomarker of SOX14 hypermethylation in cervical cancer moved forward to the validation step. The QMSP results showed that both the SOX14 methylation positive rate and methylation level were higher than those in normal cervical samples.

In a recent review that about the translational road of DNA methylation biomarkers to the clinic, Warwick J. Locke et al. mentioned that there are several diagnostic areas including primary diagnosis,triage,choice of therapy etc. for adopting the DNA methylation biomarkers [34]. Hence,we analysed the different combinations to explore the potential role of SOX14 hypermethylation in the diagnosis and prevention of cervical cancer. ROC curve analysis showed that SOX14 hypermethylation had good sensitivity (94.12%) for discrimination of cervical cancer patients that would assist for the choice of therapy.

Recently, the World Health Organization (WHO) called for action towards achieving the global elimination of cervical cancer by 2030. To achieve this goal, one of the challenges is to build effective and scaled screening strategies [35]. Although the UK National Screening Committee recommended a switch to hrHPV primary screening in 2016 [36] to substitute the lower sensitivity and subjective interpretation of cytomorphology-based methods, the lower specificity results in relatively more referrals, anxiety in false-positive women, and higher costs for the health-care system. The results of this experiment confirmed that SOX14 hypermethylation has a high specificity (81.48%) for identifying CINII, which was greatly improved compared with 14.7% for HPV detection of CINII or above [37]. It indicated a triage biomarker role of SOX14 hypermethylation followed by the primary HPV screening.

Furthermore, the detection of methylation biomarker is an objective test and can be performed on the same material used for hrHPV testing, which makes it promising for self-sampled tests. This will benefit more Chinese women who live in remote rural places [38]. As one of the principal contributors to the global burden of cervical cancer, it is very important to validate biomarkers in the Chinese population not only for effective diagnosis and prevention but also for contributing indirectly to the human methylation map.

What’s more, consistent with studies that have revealed that DNA methylation occurs in a tissue-specific, cell type-specific and stage-specific manner [39, 40],SOX14 methylation correlated with tumour histological type in our analysis. This suggests a potential capability to be a differentiated biomarker of SOX14 methylation. In contrast, studies have shown that gene methylation is associated with the sensitivity of cancer to definitive chemoradiotherapy (CRT) [41, 42], while SOX14 did not present this association. In addition, another interesting mechanism worthy to be discovered afterwards is the relationship between SOX14 methylation and its expression. It is well established that DNA methylation in the gene promoter region leading to the silencing of the corresponding gene, however, gene body CpG-methylation is not so well understood, but usually associated with higher expression of the corresponding gene [43]. The CpG-methylation site of SOX14 validated in our study located in the gene body, which showed higher methylation in cancer samples. Consistently, SOX14 had higher expression in cervical cancer tissues compared to normal tissues, even quite a few samples. It is in line with previous findings that SOX14 plays an oncogene role in cervical carcinogensis [21]. Yet, the CpG-methylation site of SOX14 validated here shows a negative correlation with expression via MEXPRESS and TCGA, which may be caused by the participation of other gene regulation factors, including histone acetylation, transcription factor, etc.

## Conclusion

Our results demonstrated that it was feasible and convenient to identify DNA methylation biomarkers through coupling with bioinformatics analysis and clinical samples. SOX14 presented hypermethylation in cervical cancers; furthermore, with the optimal cut-off value, the sensitivity and specificity in the differentiation of precancer and cancer were 94.12 and 86.46%, respectively. Additionally, the sampling method can be combined with HPV tests. Thus, scale-up screening can be performed via high-throughput detection. Nevertheless, more sustainable and large cohort clinical trials are needed to confirm the identified biomarkers worldwide to push forward the rapid development of translation medicine in the near future.

## Supplementary Information

**Additional file 1: Table S1.1.** TCGA cervical squamous cell carcinoma and endocervical adenocarcinoma patient characteristics (cancer tissues). **Table S1.2.** TCGA cervical squamous cell carcinoma and endocervical adenocarcinoma patient characteristics (Adjacent normal tissues). **Table S2.** Each CpG sites of SOX14 methylation in normal vs. CSEC. The difference value indicated that the beta value of cancer minus the normal. The adj.pval represents adjusted *P*-value.

**Additional file 2: Figure S1.** A full-length gel figure was presented for DNA Ladder PCR. Among the 38 samples of frozen tissues whose DNA fragments can be determined, 2 samples (sample 6,7) have DNA fragments with severe fragmentation or low quality and are unavailable, while the remaining 36 samples are of high quality and can be used in the following experiment.

**Additional file 3: Figure S2.** The level of SOX14 expression in different normal tissues,the samples were ordered by their expression value.

**Additional file 4: Figure S3.** Full length gel pictures for the verification results of SOX14 methylation in total 36 cervical tissues using MSP.

**Additional file 5: Figure S4.** We further repeated samples (normal1,2,5,6,7) with multiple bands from Fig S2. The PCR product for methylation was 85 bp,unmethylation was 94 bp.

**Additional file 6: FigureS5.** ROC curve for SOX14 methylation in each of the differentiated groups.

## Data Availability

The datasets used and/or analyzed during the current study are available from the corresponding author on reasonable request.

## Abbreviations

MSP: Methylation-specific PCR
QMSP: Quantitative methylation-specific PCR
ROC: Receiver operating characteristic
AUC: Area under the curve
CESC: Cervical squamous cell carcinoma and endocervical adenocarcinoma
LMICs: Low and middle-income countries
TCT: Thinprep Cytologic Test
HPV: Human papillomavirus
CIN: Cervical intraepithelial neoplasia
WHO: World Health Organization
AUC: Area Under Curve
Sen: Sensitivity
Spe: Specificity
PPV: Positive predictive value
NPV: Negative predictive value
YI: Youden index
