# Supplementary material for: SOX14 hypermethylation as a tumour biomarker in cervical cancer
Source: BMC Cancer. 2021 Jun 7;21:675. doi: 10.1186/s12885-021-08406-2 (PMC8185922; doi:10.1186/s12885-021-08406-2)
Supplement: Supplementary file 1 — Additional file 1: Table S1.1. TCGA cervical squamous cell carcinoma and endocervical adenocarcinoma patient characteristics (cancer tissues). Table S1.2. TCGA cervical squamous cell carcinoma and endocervical adenocarcinoma patient characteristics (Adjacent normal tissues). Table S2. Each CpG sites of SOX14 methylation in normal vs. CSEC. The difference value indicated that the beta value of cancer minus the normal. The adj.pval represents adjusted P-value. [file 12885_2021_8406_MOESM1_ESM.docx]

**Table S1.1** TCGA cervical squamous cell carcinoma and endocervical adenocarcinoma patient characteristics(cancer tissues)

| Pathological characteritics | | | Total case |  |
| --- | --- | --- | --- | --- |
| Age at diagnosis | |  | 46(38-57) |  |
| Figo stage |  | | 300 |  |
|  | Stage Ⅰ(n=163) | |  | 54.33% |
|  | Stage Ⅱ(n=70) | |  | 23.33% |
|  | Stage Ⅲ(n=46) | |  | 15.33% |
|  | Stage Ⅳ(n=21) | |  | 7.00% |
| Histologic diagnosis |  | | 307 |  |
|  | Cervical Squamous Cell Carcinoma(n=254) | |  | 82.74% |
|  | Adenosquamous(n=6) | |  | 1.95% |
|  | Endometrioid Adenocarcinoma of Endocervix(n=3) | |  | 0.98% |
|  | Endocervical Type of Adenocarcinoma(n=27) | |  | 8.79% |
|  | Mucinous Adenocarcinoma of Endocervical Type(n=17) | | | 5.54% |
| Tumor status |  | | 265 |  |
|  | Tumor free(n=191) | |  | 72.10% |
|  | With tumor(n=74) | |  | 27.90% |
|  |  | |  |  |

**Table S1.2** TCGA cervical squamous cell carcinoma and endocervical adenocarcinoma patient characteristics (Adjacent normal tissues)

| TCGA ID | Age | Tumor status | histologic_diagnosis |
| --- | --- | --- | --- |
| TCGA-FU-A3EO | 55 | Tumor free | Endocervical Type of Adenocarcinoma |
| TCGA-HM-A3JJ | 40 | With tumor | Cervical Squamous Cell Carcinoma |
| TCGA-MY-A5BF | 69 | Tumor free | Cervical Squamous Cell Carcinoma |

**Table S2** Each CpG sites of SOX14 methylation in normal vs. CSEC

| **PROBE** | **Normal beta value** | **CESC beta value** | **Difference value** | **adj.pval** |
| --- | --- | --- | --- | --- |
| **cg04945331** | 0.0396275 | 0.678820224 | 0.639192724 | 0.00875 |
| **cg06613095** | 0.029879 | 0.605843351 | 0.575964351 | 0.00875 |
| **cg05880330** | 0.065755067 | 0.631869875 | 0.566114808 | 0.00875 |
| **cg16428251** | 0.0818284 | 0.640894644 | 0.559066244 | 0.00875 |
| **cg27505273** | 0.023726 | 0.511674763 | 0.487948763 | 0.00875 |
| **cg22885558** | 0.079855 | 0.560076249 | 0.480221249 | 0.00875 |
| **cg08073312** | 0.0303593 | 0.487440691 | 0.457081391 | 0.009364 |
| **cg11630154** | 0.037132667 | 0.493642436 | 0.456509769 | 0.00875 |
| **cg00088130** | 0.080090667 | 0.526122479 | 0.446031812 | 0.009364 |
| **cg21368820** | 0.0793129 | 0.474123139 | 0.394810239 | 0.009364 |
| **cg04374393** | 0.211965 | 0.598228457 | 0.386263457 | 0.00875 |
| **cg10343742** | 0.15316 | 0.527859175 | 0.374699175 | 0.00875 |
| **cg14783581** | 0.093722333 | 0.452479871 | 0.358757538 | 0.016075 |
| **cg13217064** | 0.031225933 | 0.381789998 | 0.350564065 | 0.044226 |
| **cg16921083** | 0.0306813 | 0.328584944 | 0.297903644 | 0.048465 |
| **cg26869578** | 0.137964 | 0.404703818 | 0.266739818 | 0.042746 |
| **cg03811478** | 0.163099 | 0.421681414 | 0.258582414 | 0.00875 |
| **cg15787616** | 0.0397933 | 0.270765688 | 0.230972388 | 0.009364 |
| **cg03802069** | 0.131512333 | 0.297517941 | 0.166005608 | 0.05702 |

The difference value indicated that the beta value of cancer minus the normal. The adj.pval represents adjusted *P*-value.
