# Supplementary figures and images for: SOX14 hypermethylation as a tumour biomarker in cervical cancer
Source: BMC Cancer. 2021 Jun 7;21:675. doi: 10.1186/s12885-021-08406-2 (PMC8185922; doi:10.1186/s12885-021-08406-2)

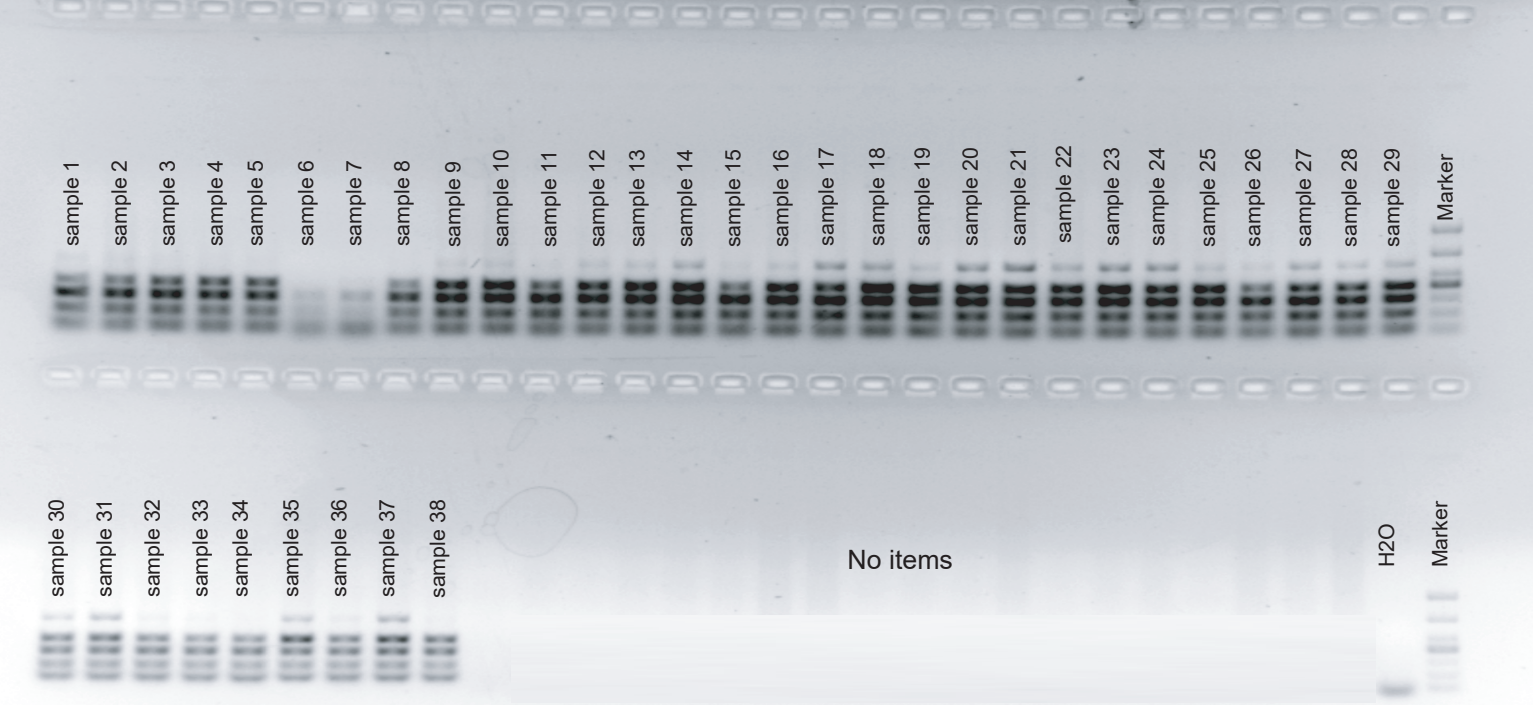

Supplement: Supplementary file 2 — Additional file 2: Figure S1. A full-length gel figure was presented for DNA Ladder PCR. Among the 38 samples of frozen tissues whose DNA fragments can be determined, 2 samples (sample 6,7) have DNA fragments with severe fragmentation or low quality and are unavailable, while the remaining 36 samples are of high quality and can be used in the following experiment. [file 12885_2021_8406_MOESM2_ESM.pdf]

GTEx dataset<sup>i</sup>

Organ

Expression

Alphabetical

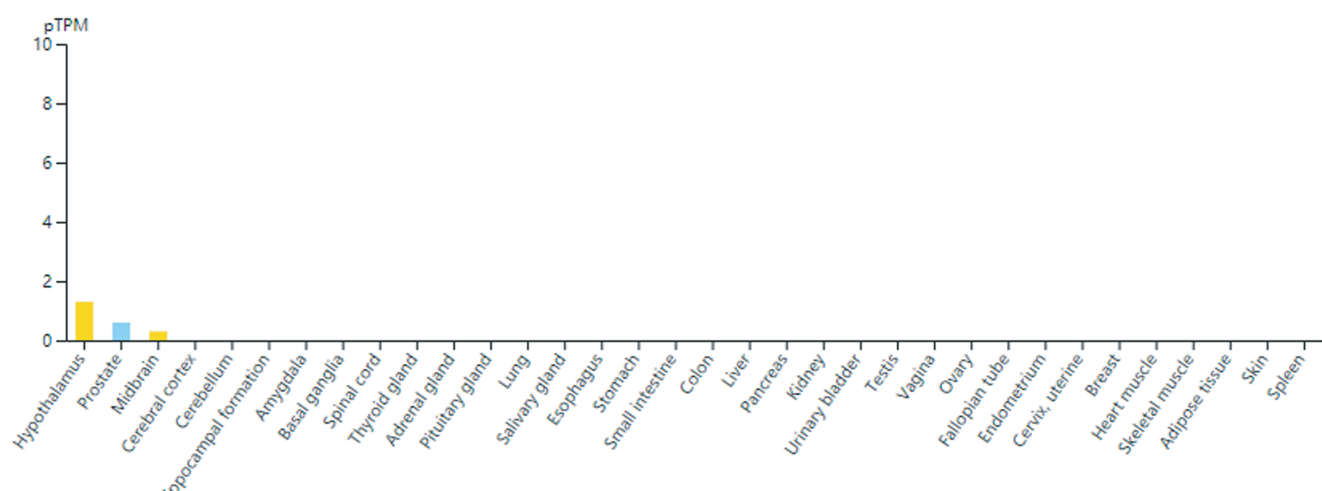

Supplement: Supplementary file 3 — Additional file 3: Figure S2. The level of SOX14 expression in different normal tissues,the samples were ordered by their expression value. [file 12885_2021_8406_MOESM3_ESM.pdf]

A

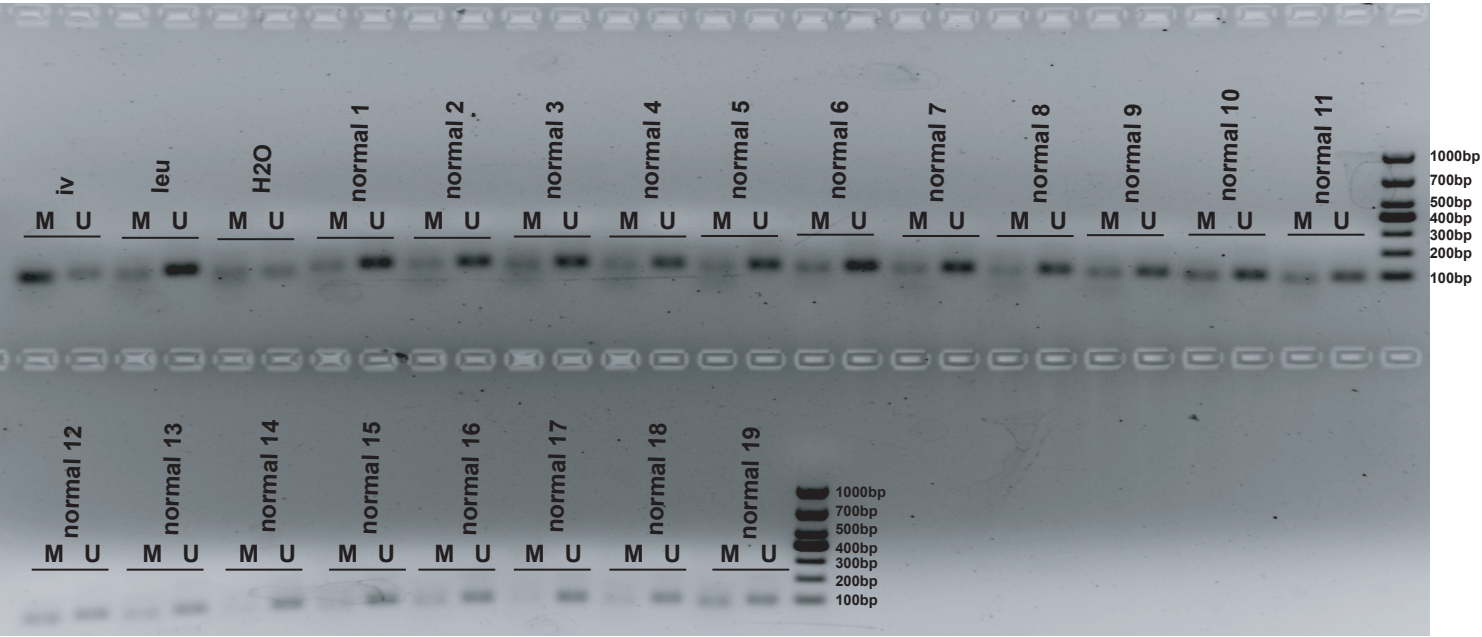

Normal

B

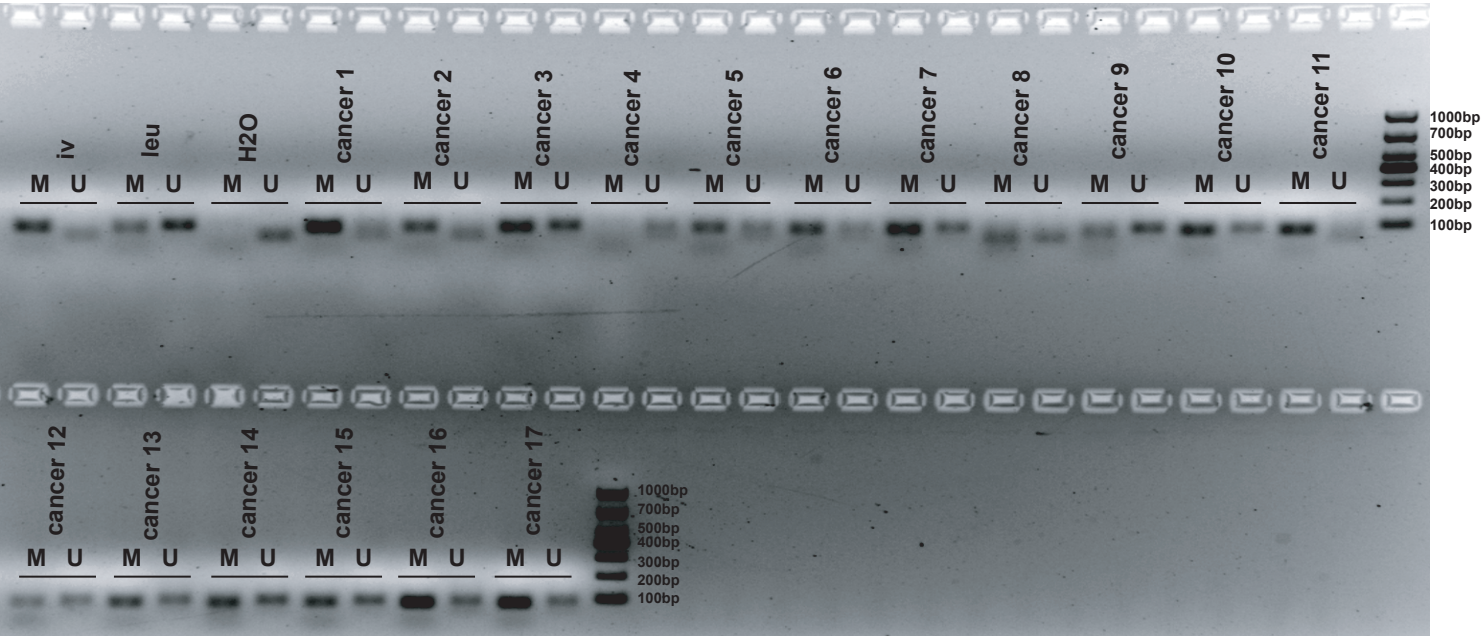

Cancer

Supplement: Supplementary file 4 — Additional file 4: Figure S3. Full length gel pictures for the verification results of SOX14 methylation in total 36 cervical tissues using MSP. [file 12885_2021_8406_MOESM4_ESM.pdf]

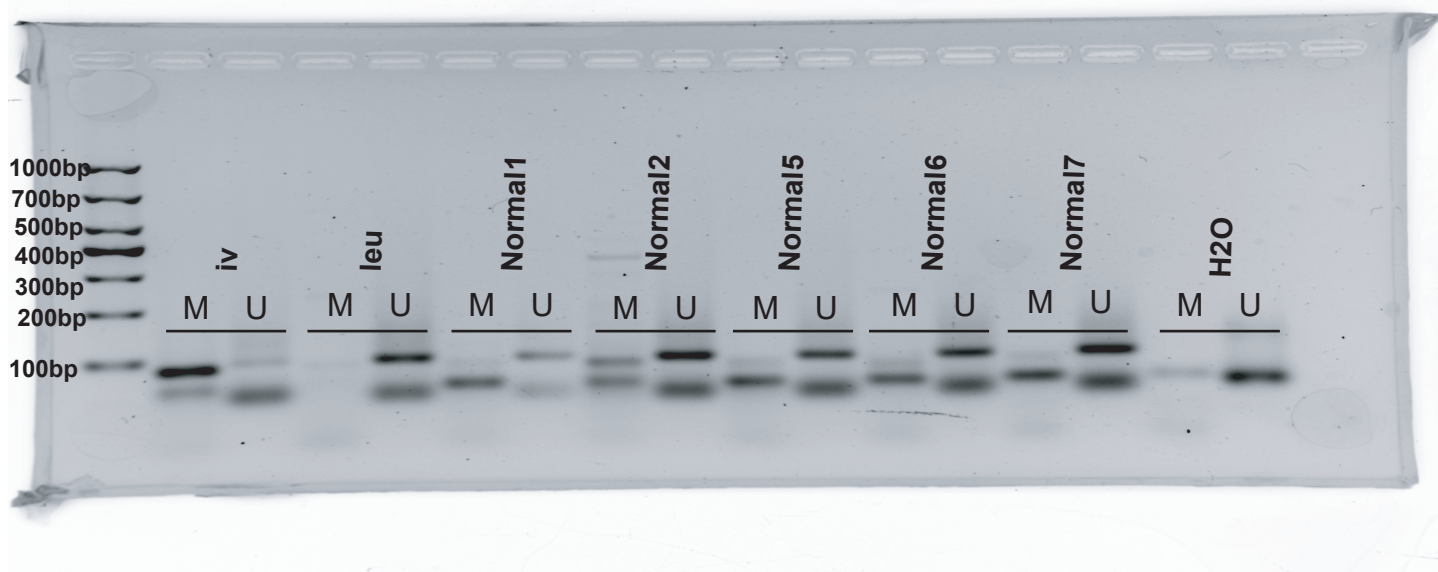

Supplement: Supplementary file 5 — Additional file 5: Figure S4. We further repeated samples (normal1,2,5,6,7) with multiple bands from Fig S2. The PCR product for methylation was 85 bp,unmethylation was 94 bp. [file 12885_2021_8406_MOESM5_ESM.pdf]

A

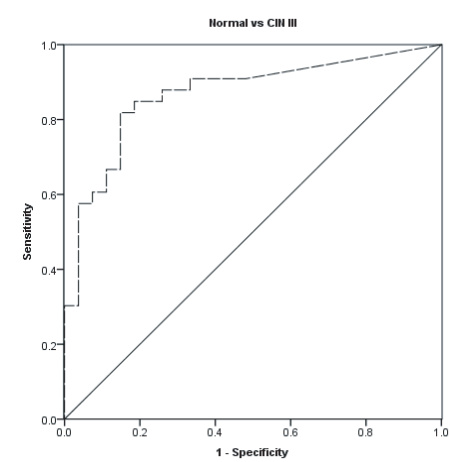

B

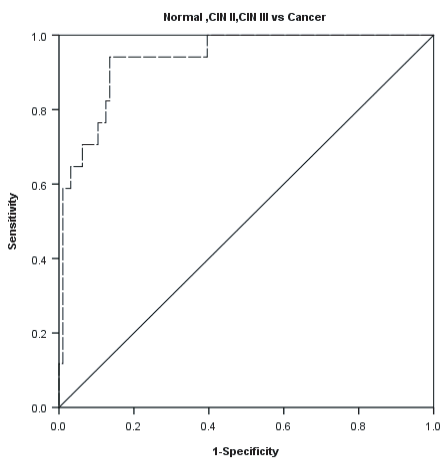

C

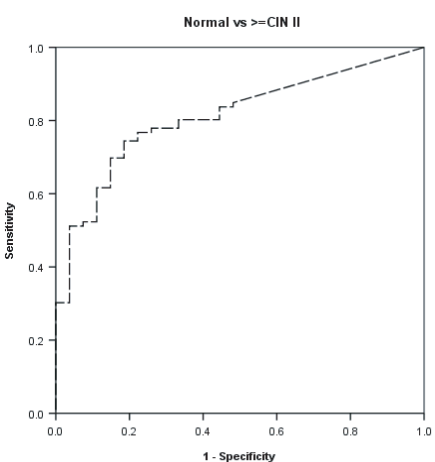

Supplement: Supplementary file 6 — Additional file 6: FigureS5. ROC curve for SOX14 methylation in each of the differentiated groups. [file 12885_2021_8406_MOESM6_ESM.pdf]
